# Crimean-Congo Hemorrhagic Fever Presented in Dengue Epidemic: A Case Report

**DOI:** 10.7759/cureus.39015

**Published:** 2023-05-14

**Authors:** Noman Salih, Khalid Saifullah Baig, Muhammad A Jan, Muhammad Ihtisham, Faizan Ahmad, Numan Ghani, Azhar Saeed, Ujala Hussain

**Affiliations:** 1 General Internal Medicine, Hayatabad Medical Complex, Peshawar, PAK; 2 Internal Medicine, Hayatabad Medical Complex, Peshawar, PAK; 3 Internal Medicine, Khyber Teaching Hospital, Peshawar, PAK; 4 Internal Medicine, Lady Reading Hospital, Peshawar, PAK; 5 Internal Medicine, Stockport NHS Foundation Trust, Manchester, GBR

**Keywords:** viral diseases, disease outbreaks, pakistan, crimean-congo, viral hemorrhagic fever

## Abstract

In Pakistan, hemorrhagic diseases, including dengue and Crimean-Congo hemorrhagic fever (CCHF), are common. Therefore, an accurate diagnosis is challenging in the early stages of sickness owing to geographic overlap and early clinical similarities between the two disorders. A 35-year-old man who had previously experienced hematemesis and high-grade fever presented to our hospital. Despite receiving supportive care for a preliminary diagnosis of dengue hemorrhagic fever, the patient's condition worsened. The results of the dengue IgM antibody test were negative. On the fourth day of admission, a qualitative polymerase chain reaction test for CCHF virus RNA was performed, and the result returned positive. All medical personnel and attendants who had contact with the patient had to receive ribavirin prophylaxis, which required significant investment in resources. Because CCHF can have long-term financial and health repercussions for contacts, including healthcare personnel in developing nations, it is essential to identify and treat it as soon as possible. It is necessary to keep track of dengue and CCHF cases more closely to develop predictors of disease diagnosis that are reasonably trustworthy, affordable, and quick. These predictors can aid in directing future choices regarding the care of similar situations. Ultimately, such an approach might result in improved cost control in environments with limited resources. Consideration should also be given to patients who receive ribavirin prophylaxis.

## Introduction

Viral hemorrhagic illnesses are a broad category of zoonotic diseases of tick-borne origin. Crimean-Congo hemorrhagic fever virus (CCHFV) is a hemorrhagic fever syndrome with a mortality rate of 30% worldwide. [1] This newly emerged entity belongs to the genus *Nairovirus* and belongs to the *Bunyaviridae* family. Transmission to humans occurs through an infected tick bite and direct contact with infected body fluids. Hyalomma ticks cause viral transmission to humans from infected animals [1]. The reticuloendothelial system is frequently affected by infection, which also activates the cytokine cascades in the body. The victims then experience increased vascular permeability, which ultimately results in hemorrhage and shock. Multiple organ systems, including the hematopoietic, neurological, and pulmonary systems, are involved as the disease progresses [2,3]. The dengue virus, on the other hand, is a member of the *Flavivirus* family and spreads via the bite of the Aedes mosquito. There are four serotypes of the virus. A person infected with one serotype has lifetime immunity to that specific serotype, but infection with any of the remaining three serotypes will have disastrous effects on that person due to a process known as "antibody enhancement" [4,5]. Infection with the dengue virus can present clinically as dengue fever (DF), dengue hemorrhagic fever (DHF), or dengue shock syndrome (DSS). Patients with DF typically present with fever, myalgia, and morbilliform rashes. Individuals with DHF also experience hemorrhagic symptoms, whereas those with DSS mostly experience circulatory failure [5]. An increase in the permeability of the vasculature is the pathophysiological driving force behind the symptoms and indications of DHF and DSS [4]. Misdiagnosis of DHF can be catastrophic because both nonspecific symptoms and some of its specific symptoms can match those of Crimean-Congo hemorrhagic fever (CCHF). Accurately distinguishing between DF and CCHF in the early stages of the illness is challenging because of the overlap in geographic distribution and similarities in the initial presentation of the two disorders. However, definitive laboratory tests, particularly those based on molecules, are not widely accessible in Pakistan. In addition, such tests are expensive and time-consuming. Therefore, patients must first be handled based only on clinical suspicion until the test results are available. When a conclusive diagnosis is available, this strategy may occasionally have significant post-diagnostic repercussions. Here, we described a confirmed case of CCHF associated with an ongoing dengue epidemic, which was initially treated with a diagnosis of DHF.

## Case presentation

**Figure 1:**
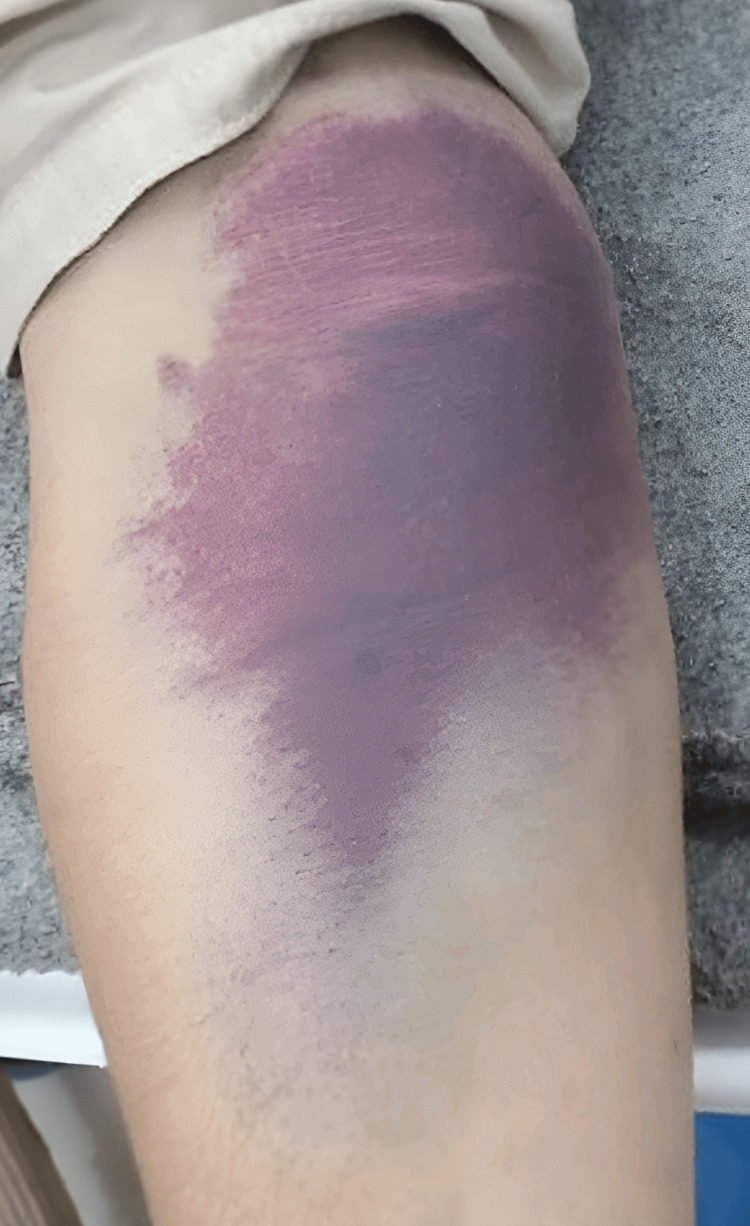
Hemorrhage on the flexor aspect of the right elbow.

The patient presented with chief complaints of fever, headache, generalized body aches, bleeding gums, and easy bruising for around seven days. Before visiting us, he received conservative treatment for a suspected dengue hemorrhagic fever at a nearby district hospital. His vital signs were heart rate of 98 bpm, blood pressure of 110/70 mmHg, respiratory rate of 19 breaths/minute, oxygen saturation of 94% on room air, and temperature of 102°F. Initial examination showed an alert, well-built man with bruising in the right upper arm (Figure 1) and bleeding gums. One day after hospitalization, GI bleeding, including fresh blood in the vomitus and stools, started. A digital rectal examination (DRE) showed fresh blood loss from the anus. Abdominopelvic ultrasonography revealed unremarkable results.

The laboratory results at admission are shown in Table 1. To rule out endemic causes like dengue and malaria, blood samples were taken for dengue serology, and two samples were taken for thick and thin smears for malarial parasites 48 hours apart. During monitoring, there was active GI bleeding for which he was given a vitamin K injection, fresh frozen plasma, platelets, and injectable procoagulants. The lab investigations revealed normal hemoglobin but his platelets were lowered. Viral markers for hepatitis A, B, and C, and Cytomegalovirus were assessed in response to raised liver enzymes; however, the results were negative. His acute phase reactants, such as ferritin, erythrocyte sedimentation rate (ESR), and C-reactive protein (CRP), were elevated. D-dimer concentration was above 1600 ng/ml (<200 = negative). The test for malarial parasites and dengue serology likewise yielded negative results. He has been performing multiple jobs but has recently started working as a butcher. The patient was isolated, and contact precautions were advised to the personnel as well as the family members due to a suspicion of CCHF due to his occupation, clinical symptoms, and negative results for other endemic diseases. The patient and their contacts were started on prophylactic ribavirin. At the Khyber Medical University in Peshawar, Pakistan, the polymerase chain reaction (PCR) assay for the Congo virus was conducted. The assay's specificity was determined as 100% and returned positive; hence, the diagnosis was verified. The patient was continued on ribavirin medication after confirmation of his CCHF. His bleeding stopped with conservative measures and after stabilization, he was discharged on ribavirin, paracetamol, as-needed vitamin K injection, and injectable procoagulants. He was asked to come for a follow-up after completing his ribavirin medications or before that in case his condition gets deteriorated. On a follow-up visit after three weeks, he was doing fine except for the bruise on his arm. Luckily, none of the contacts suffered from CCHF perhaps due to timely precautionary measures and ribavirin administration.

**Table 1:** Hematological investigations on admission. Abbreviations: ALP, alkaline phosphatase; ALT, alanine aminotransferase; AST, aspartate aminotransferase; BUN, blood urea nitrogen; Cr, creatinine; Hb, hemoglobin; INR, international normalized ratio; PLT, platelets; PT, prothrombin time; PTT, partial thromboplastin time; WBC, white blood cells.

Laboratory test	Reference range	At admission
WBC (µ/l)	4000–10,000	9750
Hb (gr/dl)	13.5–17.5	15.6
PLT (/µl)	150,000–450,000	20,100
PTT (sec)	30–45	50
PT (sec)	12–14	16.4
INR	1–1.19	1.3
BUN (mg/dl)	18-45	22
Cr (mg/dl)	0.6–1.2	0.97
AST (IU/L)	Up to 37	134
ALT (IU/L)	10–50	250
ALP	40–129	301

## Discussion

After the dengue virus, CCHF is the second most prevalent viral hemorrhagic fever, and Hyalomma ticks viruses serve as both its vector and reservoir. Many countries in Southeast Europe, Africa, and Asia are endemic to this infection [6]. Patients with this complex viral disease may have mild clinical symptoms or develop deadly complications. Four phases make up the clinical course of CCHF infection: incubation, pre-hemorrhagic, hemorrhagic, and healing phases. Clinical symptoms in the pre-bleeding stage after a variable incubation phase include an abrupt onset of fever, chills, headache, abdominal discomfort, and myalgia, which typically lasts for about a week. Accordingly, indications of blood problems such as petechiae and disseminated intravascular coagulation start to appear after three to six days in severe cases [7]. As a result of the virus's suppression of the host's immune system, the virus proliferates quickly, and the host's vascular system is dysregulated, which contributes to the disease's pathogenesis. Intravascular coagulation and multi-organ failure, its most devastating complications, have been linked to inflammatory mediators, which then activate endothelial and intrinsic coagulation cascades [8].

Many laboratory results have been reported as prognostic indicators, including hemoglobin, platelet count, prothrombin time, international normalized ratio, activated partial thromboplastin time, aspartate aminotransferase, and alanine aminotransferase. The presence of CCHF is typically suspected based on clinical signs and symptoms, and epidemiological and laboratory criteria. Early diagnosis is essential for the treatment and avoiding transmission of infection. Reverse transcription-polymerase chain reaction (RT-PCR) or a four-fold increase in positive CCHFV IgM or specific IgG levels are used to confirm the diagnosis. In the second week of the illness, these serological tests are useful [9,10]. Ribavirin is the only antiviral medication that may help treat CCHFV, especially in the early phases of therapy [11].

In peripheral areas of Khyber Pakhtunkhwa, CCHF is a rare cause of tick-borne viral infection. The first diagnosed human infection case in the region was reported in 1999, and since then, the incidence of CCHF has somewhat increased. In Pakistan, the virus is primarily transmitted through contact with livestock. As a result, the number of cases significantly increases every year following the eve of Eid al-Adha. The first case of CCHF in Pakistan was identified in 1976, and 14 more cases were reported in the next 34 years, i.e., between 1976 and 2010 [12]. According to the National Institute of Health in Islamabad, Pakistan, 356 cases of CCHF were diagnosed between January 2014 and May 2020, with a 25% mortality rate. Of these cases, 38% were from Baluchistan, 23% from Punjab, 19% from Khyber Pakhtunkhwa, 14% from Sindh, and 6% from Islamabad [13]. Zohaib et al. discovered a 2.7% seroprevalence of CCHF, with a higher incidence among rural residents, possibly due to increased interaction with animals [14].

We described a case of confirmed CCHF that had symptoms like those of DHF at first and was treated accordingly, but afterward, gastrointestinal bleeding manifested. The patient initially presented with a high fever, headache, and generalized body aches. The diagnosis of CCHF was made following admission to our hospital, a detailed review of the history, and test results. Early signs of CCHF include fever, generalized body aches, bleeding gums, headache, weakness, and diarrhea, symptoms that are shared with DHF. Therefore, it could prove exceedingly dangerous and problematic to misdiagnose CCHF as DHF. Clinical and epidemiological findings are important strategies for diagnosing CCHF [15].

## Conclusions

In summary, our case represents a complex clinical scenario in which the patient was initially suspected of having DHF, a disease that does not require specific measures such as contact isolation or the use of oral antiviral agents. However, the diagnosis of CCHF was established on the fourth day of admission. Considerable resources were spent not only on tracking contacts who had been exposed but also on administering post-exposure prophylaxis to them. The diversion of hospital resources toward these measures represents a significant degree of disruption to prevent the spread of the disease. In addition, CCHF cases may generate considerable stress and fear among hospital staff and other contacts. CCHF-related deaths among healthcare workers have been reported in Pakistan on a few occasions. Therefore, we need to recognize CCHF at an early stage. However, this is not always possible. Improved education of healthcare workers and better surveillance of DF and CCHF should be ensured in our region so that we can devise reasonably reliable, cost-effective, and prompt predictors of disease diagnosis based on the collected data. As this case demonstrates, one of the biggest challenges faced is the early recognition of CCHF in dengue-endemic areas.
